# Effect of isolation techniques on the characteristics of pigeon pea (*Cajanus cajan*) protein isolates

**DOI:** 10.1002/fsn3.539

**Published:** 2017-11-12

**Authors:** Monilola K. Adenekan, Gbemisola J. Fadimu, Lukumon A. Odunmbaku, Emmanuel K. Oke

**Affiliations:** ^1^ Department of Food Technology Moshood Abiola Polytechnic Ojere Abeokuta Nigeria; ^2^ Department of Food Science and Technology Federal University of Agriculture Abeokuta Nigeria; ^3^ Department of Food Science and Human Nutrition King Saud University Riyadh Saudi Arabia

**Keywords:** Functional properties, isolation techniques, pigeon pea, proximate composition, antinutritional property

## Abstract

In this study, the effect of different isolation techniques on the isolated proteins from pigeon pea was investigated. Water, methanol, ammonium sulfate, and acetone were used for the precipitation of proteins from pigeon pea. Proximate composition, and antinutritional and functional properties of the pigeon pea flour and the isolated proteins were measured. Data generated were statistically analyzed. The proximate composition of the water‐extracted protein isolate was moisture 8.30%, protein 91.83%, fat 0.25%, ash 0.05%, and crude fiber 0.05%. The methanol‐extracted protein isolate composition was moisture 7.87%, protein 91.83%, fat 0.17%, and ash 0.13%, while crude fiber and carbohydrates were not detected. The composition of the ammonium sulfate‐extracted protein isolate was moisture 7.73%, protein 91.73%, fat 0.36, ash 0.13%, and crude fiber 0.67%. The acetone‐extracted protein isolate composition was moisture 8.03%, protein 91.50%, ash 0.67%, and fat 0.30%, but crude fiber and carbohydrates were not detected. The isolate precipitated with ammonium sulfate displayed the highest foaming capacity (37.63%) and foaming stability (55.75%). Isolates precipitated with methanol and acetone had the highest water absorption capacity (160%). Pigeon pea protein isolates extracted with methanol and ammonium sulfate had the highest oil absorption capacity of 145%. Protein isolates recovered through acetone and methanol had the highest emulsifying capacity of 2.23% and emulsifying stability of 91.47%, respectively. The proximate composition of the recovered protein isolates were of high purity. This shows the efficiency of the extraction techniques. The isolates had desirable solubility index. All the isolation techniques brought significant impact on the characteristics of the isolated pigeon pea protein.

## 1 INTRODUCTION

Pigeon pea (*Cajanus cajan*) is a dry leguminous crop cultivated for food in Nigeria. Pigeon pea seeds have a growing season of 6–9 months, and are either harvested dry and used mainly in dal soup, or harvested earlier and eaten as a vegetable. The protein content of commonly grown pigeon pea cultivars ranges between 17.9 and 24.3 g/100 g (Salunkhe, Chavan, & Kadam, 1986) for whole grain samples and between 21.1 and 28.1/100 g for split seed. Wild species of pigeon pea have been found to be a very promising source of high protein, and several high‐protein genotypes have been developed with protein contents as high as 32.5% (Singh & Bains, 1988). The high‐protein genotypes also contain significantly higher (about 25%) sulfur containing amino acids, namely, methionine and cystine (Singh & Bains, 1988). Pigeon pea is nutritionally similar to cowpea *(Vigna unguiculata)* and can be used in much the same way (Henshaw, Oluwatola, & Aroyewun, 1999). Pigeon pea protein contains amino acids similar to that of soybean (Singh & Bains, 1988).

Protein is a macronutrient composed of amino acids, and is necessary for proper growth and function of the human body. The protein isolate is the purest form of protein which makes a great dietary supplement, and it is beneficial for physical strength performance and weight management (Kate, 2011). The consumption of plant protein isolate with special reference to legume is beneficial (Nunes, Raymundo, & Sousa, 2006). It is recognized that protein isolate offers immense possibility in the development of new class of formulated food.

The demand for proteins in the world is increasing and so more food protein is required for both conventional and new source of proteins (Abdel‐Rahman, Eltayeb, Azza, & Feria, 2011). Being a cheap source of proteins for low‐income group of the population, legumes are commonly used as a substitute for meat and play a significant role in alleviating the protein–energy malnutrition (Mateos‐Aparico, Redondo, Villanueva‐Suarez, & Zapata‐Revilla, 2008). Lack of sufficient protein in nutrition of large percentage of people in developing countries is a major setback for human development (Yemisi & Kayode, 2007). Malnutrition is a major nutritional problem in the developing world with specific maladies, like kwashiorkor and marasmus especially in children, and in adults, it results in poor health and reduced capacity (Mateos‐Aparico et al., 2008). These existing problems of food security and malnutrition coupled with escalating population and a high cost of animal‐based food supplies led to identify and incorporate unconventional protein sources to enrich traditional formulation and conventional food. The protein starved condition of the inhabitants in tropical Africa and other parts of the world could be improved greatly by widespread use of edible legumes since they are an important source of the essential amino acids (Alsohaimy, Sitouhy, & El‐Masry, 2007).

In spite of the potential, pigeon pea appears to be an underutilized legume in the subregions of West Africa, especially in Nigeria. At present, the cultivation is gradually being left to extinction. In order to prevent extinction and to increase the pigeon pea production and utilization, one of the approaches is to exploit its protein component.

A good isolation technique ensures highly purified protein. An alkaline isoelectric precipitation method is the commonly applied isolation method in the industry (Ragab, Babiker, & Eltinay, 2004). However, information on the effect of other isolation techniques, methanol precipitation method, water extraction method, ammonium sulfate extraction method, and acetone precipitation method, on the characteristics of pigeon pea protein isolates is still scarce. The objective of this work was to isolate proteins using the aforementioned methods from Nigerian pigeon pea and to establish the potential for industrial application.

### 1.1 Material and preparation of pigeon pea flour

Pigeon pea (*Cajanus Cajan*) seeds used for this project work were purchased from Oje market, Ibadan, Nigeria. Pigeon pea seeds were sorted, and cleaned to remove immature seeds, stones, dirt, stalk, and unwanted particles. The size of cleaned pigeon pea seeds were reduced to facilitate dehulling and then milled in an attrition mill (Model: All Asiko, Nigeria) until a uniform fine powder was obtained. The flour was stored and labeled accordingly (A and B) in a polyethylene bag until further use.

### 1.2 Preparation of protein solution

The protein extraction was carried out using the method described by Jang, Wang, and Zhang (2005) and Sanchez‐Vioque (1999). Pigeon pea seeds (750 g) were suspended in 200 ml of distilled water at room temperature. The pH was adjusted to 11 with NaOH. The pH was maintained for 30 min at room temperature followed by agitation until no foaming was observed. The recovered suspension was centrifuged using a Hitachi high‐speed refrigerated centrifuge (model: Himac CR22N) at 6,000 rpm, 20°C for 30 min. The supernatant was stored at 4°C in a refrigerator until further use. The same procedure was performed for the second sample.

### 1.3 Protein isolation methods

Four different isolation methods, methanol precipitation method, water extraction method, ammonium sulfate extraction method, and acetone precipitation method, were used for the preparation of pigeon pea protein isolate. Protein isolation using water was done using the method described by Berghout et al., (2014) the methanol precipitation method was carried out using the method described by Schwenke (2001), and ammonium sulfate extraction and acetone precipitation methods were carried out as described by Sathe and Salunkhe (1981).

### 1.4 Analyses of the proximate composition, antinutritional factors, and functional properties of the samples

The samples were evaluated for proximate composition, antinutritional and functional properties, protein solubility, nutritional properties, and amino acid profiles. The proximate composition of the samples was determined using standard AOAC (2003) methods. The phytic acid content of the samples was determined using the methods described by Wheeler and Ferrel (1971), trypsin inhibitor using Arntfield, Ismound, and Murray (1985) method, and cyanogenic glucoside content using acid titration method as described by AOAC (2003).

The water and oil absorption capacities of the samples were determined using the method described by Sosuiski, Humbert, Bui, and Jones (1976), emulsion capacity and stability using the Naczk, Diosady, and Rubin (1985) method, bulk density using the modified method of Okaka and Potter (1977), and foaming capacity and stability using the method described by Lin, Humbert, and Sosuiski (1974).

The protein solubility was determined using the method described by Morr et al. (1985). Also, the nutritional properties of the samples were determined using the rat assay. The diet preparation was done using the method described by Fernandez‐quintela, Del barrio, Macarulla, and Martinez (1998). The amino acid analysis was done using high‐performance liquid chromatography (HPLC) as described by Igor, Sasa, Dragan, Sandra, & Biljana, 2012.

### 1.5 Statistical analysis

Experimental data were subjected to an analysis of variance (ANOVA) and the means separated by a Duncan's new multiple range test (DMRT) using the SPSS 11.0 version (Michigan State University, East Lansing, MI) at a significance level of .05.

## 2 RESULTS AND DISCUSSIONS

### 2.1 Effect of isolation techniques on the proximate and antinutritional composition of pigeon pea protein flour and protein isolates

The result of the proximate composition of pigeon pea flour and protein isolate samples are presented in Table 1. The proximate composition of the pigeon pea flour sample and the different isolate were significantly different (*p *<* *.05). The chemical composition is a simple and convenient way illustrating the purity of the protein isolate where higher protein content and lower content of other components, fat, ash, carbohydrate, and fiber, are highly desirable. However, the result shows that the recovered protein isolates contained neither crude fiber nor carbohydrate. The percentage of fat (0.17–0.36%) and ash (0.05–0.67%) were also significantly (*p *<* *.05) low. This is desirable as it further indicates the purity of the recovered protein isolates The high value of protein (91.83%) recovered was similar to 90.65 + 0.25% that obtained from pigeon pea protein isolate from another cultivar, which was isolated through alkaline isoelectric precipitation technique (Olawunmi, Ojukwu, & Eboh, 2012), and favorably higher than 86.9% for mucuna bean protein isolate as reported by Mugendi et al. (2010). Likewise, the value was higher than the protein content of rapeseed protein isolate (82.0%) and lower than that of soy protein isolate (96.0%) (Kinsella, 1976). A high percentage of protein recovered shows that water, methanol, ammonium sulfate, and acetone are good precipitants of protein from the examined food system. This high content of protein isolate showed that the isolate could be incorporated into foods like ice cream, baked products, and infant food for enrichment purpose to increase the protein pool.

**Table 1:** Proximate composition of pigeon pea flour and protein isolates

	Moisture (%)	Protein (%)	Fat (%)	Ash (%)	Fiber (%)	Carbohydrate (%)
PPF	6.70 ± 0.20^a^	19.70 ± 6.20^a^	1.40 ± 0.0^d^	3.50 ± 0.10^d^	3.20 ± 0.20^b^	60.40 ± 0.00
WEPI	8.30 ± 0.00^d^	91.35 ± 0.21^b^	0.25 ± 0.7^b^	0.05 ± 0.07^a^	0.05 ± 0.07^a^	ND
MEPI	7.87 ± 0.06^c^	91.83 ± 0.06^b^	0.17 ± 0.06^a^	0.13 ± 0.06^b^	ND	ND
ASEPI	7.73 ± 0.06^b^	91.73 ± 0.12^b^	0.36 ± 0.06^c^	0.67 ± 0.06^c^	ND	ND
ACEPI	8.03 ± 0.06^c^	91.50 ± 0.10^b^	0.30 ± 0.10^c^	0.16 ± 0.06^b^	ND	ND

All values are means of triplicates determinations ± standard deviation (SD). Means within the same column with different superscript are significantly different (*p *< .05).

WEP, water‐extracted pigeon pea protein isolate; MEPI, methanol–extracted pigeon pea protein isolate; ASEPI, ammonium sulfate‐extracted pigeon pea protein isolate; ACEPI, acetone‐extracted pigeon pea protein isolate; PPF, pigeon pea flour; ND, not detected.

The result of antinutritional composition of flour and protein isolate samples were presented in Table 2. There were significant difference (*p *<* *.05) between the antinutritional content of the pigeon pea flour (2.70 mg/100 g for tannin and 4.60 mg/100 g for phytate) and the different protein isolates (0.80–1.07 mg/100 g for tannin and 1.63–3.17 mg/100 g for phytate). Antinutrients are natural or synthetic compounds that interfere with the absorption of nutrients. They inhibit the optimum utilization of nutrients, and have been reported to impair the bioavailability of protein. Antinutritional contents of pigeon pea flour sample were tannin 2.70 ± 0.5 mg/100 g, phytate 4.60 + 0.0 mg/100 g, and trypsin inhibitor 26.8 ± 0.0 mg/100 g. The result of antinutritional composition of the flour in comparison with the protein isolate shows that there were drastic reduction in the levels of tannin and phytate; however, trypsin inhibitor and cyanogenic glucoside were not detected in the protein isolate. The low levels of tannin and phytate, and nondetection of trypsin inhibitor and cyanogenic glucoside in all the prepared pigeon pea protein isolate samples compared to the flour are an indication of efficiency of all the isolation methods adopted. However, such low levels of tannin ensure noninhibition of digestive enzyme activity by tannin when the isolates are consumed. Phytic acid reduces the bioavailability of some essential minerals (Duhan, Chuhan, Punia, & Kapoor, 1989), and could form complexes with proteins (protein–phytate complexes) and chelates essential dietary minerals (such as iron, calcium, and magnesium), thus, decreasing their utilization (Kratzer, 1965).

**Table 2:** Antinutiritional composition of pigeon pea flour and protein isolates

	Tannin (mg/100 g)	Phytate (mg/100 g)	Trypsin inhibitor (TUI/100 g)	Cyanogenic glucoside (mg/100 g)
PPF	2.70 ± 0.50^c^	4.60 ± 0.0^d^	26.8 ± 0	ND
WEPI	0.87 ± 0.06^a^	2.50 ± 0.00^b^	ND	ND
MEPI	0.97 ± 0.06^a^	1.63 ± 0.15^a^	ND	ND
ASEPI (R)	1.07 + 0.16^b^	3.17 + 0.29^c^	ND	ND
ACEPI (R)	0.80 + 0.00^a^	2.00 + 0.00^b^	ND	ND

All values are means of triplicates determinations ± standard deviation (SD). Means within the same column with different superscript are significantly different (*p *< .05).

WEP, water‐extracted pigeon pea protein isolate; MEPI, methanol‐extracted pigeon pea protein isolate; ASEPI, ammonium sulfate‐extracted pigeon pea protein isolate; ACEPI, acetone‐extracted pigeon pea protein isolate; PPF, flour pigeon pea flour; ND, not detected.

### 2.2 Effect of isolation techniques on the functional properties of pigeon pea protein flour and protein isolates

The functional properties of the pigeon pea flour and protein isolates are presented in Table 3. The result of functional properties of the pigeon pea flour was significantly different (*p *<* *.05) from that of the protein isolates. Loose (0.43–0.45 g/ml) and packed (0.62–0.64 g/ml) bulk density of the pigeon pea protein were not significantly different (*p *>* *.05). However, all the pigeon pea protein isolate samples were significantly different in terms of other functional properties measured. Functional properties have been defined as the physiochemical properties that give information on how protein behave in the food system, either as a processing aid or as direct contributor of product attribute (Wilding, 1974). Functional properties determine the application and use of food materials for various food products. Bulk density is a reflection of the load the samples can carry if allowed to rest directly on one another. It is generally affected by the particle size and the density of the material (Okpala and Mamah, 2001). The values of bulk density (loose and packed) determined for all the pigeon pea protein isolates were low, and were not significantly different (*p *>* *.05) from one another and that of the flour sample. In fact, such low bulk density makes the protein isolates important in relation to packaging and would also enhance in the formulation of weaning foods (Eneche & Owheruo, 2005). Foam is produced when air is injected into a liquid and entrapment in the form of bubbles takes place. The foaming capacity (33.23–35.37%) and stability (52.33–55.73%) displayed by all the pigeon pea protein isolates in the present work were higher than that displayed by the counterpart flour samples, showing the capability of all the isolation techniques applied in the present work. However, pigeon pea protein isolates precipitated with ammonium sulfate displayed the highest foaming capacity (37.63%) and foaming stability (55.75%) (Table 3). These foaming properties suggest that the produced isolates studied in this work may be attractive in products like cakes or whipping topping, where foaming characteristics are important (Kinsella, 1976). However, Grahams and Phillips (1976) linked good foamability with flexible protein molecules that can reduce surface tension while highly ordered globular protein, which is relatively difficult to surface denature give low foamability. Therefore, one may suggest that the pigeon pea protein is a highly flexible protein. Water absorption characteristics represents the ability of a product to associate with water under conditions where water is limiting, for example, dough and pastes (Giami, Bekebain, & Emelike, 1992). All the isolation techniques conferred great improvement to pigeon pea protein isolates. The water absorption values of the isolates were higher than that of the counterpart flour sample. However, pigeon pea protein isolates precipitated with methanol and acetone had the highest water absorption (160%) (Table 3). The water absorption reported in the present study suggest that the prepared and examined protein isolates may be used in the formulation of some foods such as sausage, dough, processed cheese, soups, comminuted meat, baked products and doughnut, where handling characteristics, mouth feel, and textural quality of food are affected by water incorporation (Olaofe, Arogundade, Adeyeye, & Falusi, 1998; Oshodi & Ekperigin, 1989). The same trend was observed in the result for oil absorption. Pigeon pea protein isolates extracted with methanol and ammonium sulfate had the highest oil absorption (145%) (Table 3). The oil absorption capacity has been attributed to the physical entrapment of oil. This is important since oil acts as a flavor retainer and increases the mouth feel of foods (Eke & Akobundu, 1993). The emulsifying activity of the protein isolate has shown that protein isolates recovered through acetone and methanol had the highest emulsifying capacity (2.23%) and emulsifying stability (91.47%), respectively (Table 3). The values reported for the emulsifying stability by Masood and Rizwana (2010) was higher (83.30%) compared with protein isolates of another pigeon pea cultivars. The emulsifying properties of the pigeon pea protein isolates make them an useful additive for stabilization of fat emulsions in the production of sausages, soups, and cakes (Altschul, 1974). The solubility profile of a protein provides some insight into the extent of denaturation or irreversible aggregation and precipitation, which might have occurred during the isolation process. It also gives an indication of the types of foods or beverages into which the protein could be incorporated. Factors such as concentration, pH, ionic strength, and the presence of other substance influence the solubility of protein (Yemisi & Kayode, 2007). The whole protein solubility was clearly dominated by the behavior of the globulins. Protein isolates precipitated with water and methanol had the highest solubility with value 97.93% and 97.13%, respectively. However, it has been shown that all the techniques brought great improvement in the solubility of the pigeon pea protein isolates (Table 3). Protein solubility has been reported as an important prerequisite for food protein functional properties, and it is a good index of potential applications of proteins in food systems (Kinsella, 1976).

**Table 3:** Functional properties of pigeon pea protein flour and isolate

	Bulk density (loose) (g/cm^3^)	Bulk density (packed) (g/cm^3^)	Foaming capacity (%)	Foaming stability (%)	Water absorption capacity (%)	Oil absorption capacity (%)	Emulsifying capacity (%)	Emulsifying stability (%)	Solubility index (%)
PPF	0.40 ± 0.00^a^	0.60 ± 0.00^a^	18.20 ± 0.00^a^	25.30 ± 1.10^a^	125.00 ± 0.00^a^	131.6 ± 0.60^a^	0.40 ± 0.00^a^	0.40 ± 0.080^a^	12.10 ± 0.02^a^
WEPI	0.43 + 0.0^a^	0.62 + 0.0^a^	33.3 + 0.2^b^	54.57 + 0.15^d^	155.00 ± 0.00^c^	140.00 ± 0.00^b^	1.40 ± 0.00^b^	91.20 ± 0.10^c^	97.03 ± 0.10^c^
MEPI	0.43 + 0.0^a^	0.62 + 0.0^a^	35.37 + 0.15^c^	53.83 + 0.15^c^	160.00 ± 0.02^d^	145.00 ± 0.02^c^	1.40 ± 0.02^b^	91.47 + 0.12^c^	97.13 ± 0.12^c^
ASEPI	0.45 ± 0.01^a^	0.64 ± 0.01a	35.10 ± 0.36^c^	55.73 ± 0.70^d^	151.67 ± 2.89^b^	145.00 ± 0.00^c^	2.17 ± 0.06^c^	89.10 ± 0.36^b^	90.70 ± 2.20^b^
ACEPI	0.45 ± 0.00^a^	0.62 ± 0.00^a^	33.23 ± 0.56^b^	52.33 ± 0.31^b^	150.00 ± 0.00^b^	140.00 ± 0.00^b^	2.23 ± 0.06^c^	90.43 ± 0.15^c^	92.63 ± 0.01^b^

All values are means of triplicates determinations ± standard deviation (SD). Means within the same column with different superscript are significantly different (*p *< .05).

WEP, water‐extracted pigeon pea protein isolate; MEPI, methanol‐extracted pigeon pea protein isolate; ASEPI, ammonium sulfate‐extracted pigeon pea protein isolate; ACEPI, acetone‐extracted pigeon pea protein isolate; PPF, pigeon pea flour.

### 2.3 Effect of isolation techniques on the amino acid composition, and nutritional properties of pigeon pea protein flour and protein isolates

Results of amino acid composition of pigeon pea flour and counterpart protein isolates have shown that there were no apparent difference between the pigeon pea flour and the counterpart protein isolates in terms of amino acid composition, indicating that the process of isolation techniques applied in this work was a gentle procedure that did not affect the amino acid profile. This also shows that the nutritional quality of the starting material (flour) in terms of amino acid profile was retained and maintained. It has been shown from the result that amino acid composition of pigeon pea protein isolates are well balanced and suitable for human consumption as a source of protein for nutrition (Table 4). The result of amino acid composition has shown that all isolation techniques were suitable for the preparation of protein isolate from the pigeon pea. According to Vernon and Peter (1994), glycine together with alanine, proline, arginine, serine, isoleucine, and phenylalanine promotes growth and ensure tissue healing.

**Table 4:** Amino acid composition of (g/100 g protein) of pigeon pea flour and counterpart

Amino acid	PPF	WEPI	MEPI	ASEPI	ACEPI
Phenylanine	9.46	10.50	10.40	10.50	10.30
Valine	11.13	11.50	11.70	11.00	12.01
Theronine	7.16	8.30	8.20	8.40	8.71
Tryptophan	2.18	3.91	3.90	2.75	3.50
Isoleucine	6.33	6.50	6.40	6.30	6.50
Methionine	1.98	2.00	2.14	2.17	2.14
Histidine	1.91	1.81	1.81	1.85	1.91
Arginine	3.18	4.20	4.10	4.40	4.30
Lysine	5.74	6.54	5.00	6.00	6.53
Leucine	9.27	10.01	11.05	10.50	10.04
Cystine	4.66	5.06	4.06	5.07	5.12
Alanine	2.56	3.56	3.70	3.65	3.12
Tyrosine	7.11	7.12	6.95	7.00	7.00
Glycine	10.32	11.12	11.14	11.12	11.14
Serine	5.29	4.13	4.17	4.35	4.30
Aspartic acid	3.32	3.35	3.45	3.61	3.11
Glutamic acid	1.67	1.90	1.95	1.95	1.93
Asparagine	3.77	4.00	4.00	4.23	4.13
Proline	2.48	2.50	3.00	2.50	2.44

All values are means of triplicates determinations ± standard deviation (SD). Means within the same column with different superscript are significantly different (*p *< .05).

PPF, pigeon pea flour; WEP, water‐extracted pigeon pea protein isolate; MEPI, methanol‐extracted pigeon pea protein isolate; ASEPI, ammonium sulfate‐extracted pigeon pea protein isolate; ACEPI, acetone‐extracted pigeon pea isolate.

Results of the rat assay for casein, pigeon pea flour, and counterpart protein isolates has shown that in vivo digestibility values of pigeon pea flour were significantly low (*p *<* *.05) compared to that of casein as shown (Table 5). The result of the rat assay has shown that there were significant difference (*p *<* *.05) between the values documented for casein and the pigeon pea products. The rat assay of the pigeon pea flour was also significantly different (*p *<* *.05) from that of the counterparts protein isolate samples. Likewise, the pigeon pea protein isolates also differ from one another in terms of the rat assay profile. While the rat assay profile of all the pigeon pea protein isolates compared favorably with that of casein except on the value of protein efficiency ratio (PER) (Table 5). The higher digestibility of the pigeon pea protein isolates may be linked to reduce and eliminate antinutritional factors of the protein isolates. All the protein isolates had good and biological desirable value, net protein utilization and true digestibility. This shows the efficiency of all the isolation techniques applied. These desirable attributes of the pigeon pea protein isolates make them a good source of protein fortificant in a variety of food products to combat protein deficiency in many parts of the world, particularly developing countries. This observation agrees with other studies on other vegetable protein (Ragab et al., 2004). However, PER values has shown that all the protein isolates were in the category of intermediate quality.

**Table 5:** Rat assay of pigeon pea flour and the counterpart protein isolates

Samples	NB (g)	BV (%)	NPU (%)	TPD	PER
Casein	0.71 ± 0.06^c^	99.5 ± 1.66^c^	99.3 ± 0.34^c^	91.5 ± 0.51^b^	2.9 ± 4.3^b^
PPF	0.44 ± 0.01^a^	82.3 ± 1.2^a^	83.4 ± 0.15^a^	80.1 ± 0.0^a^	1.5 ± 3.3^a^
WEPI	0.66 ± 0.02^b^	90.0 ± 0.0^b^	93.0 ± 0.1^b^	93.1 ± 0.1^b^	1.4 ± 1.0^a^
MEPI	0.67 ± 0.07^b^	91.0 ± 0.5^b^	92.0 ± 0.5^b^	95.2 ± 0.3^c^	1.7 ± 0.1^a^
ASEPI	0.65 ± 0.01^b^	92.0 ± 0.7^b^	94.0 ± 0.3^b^	96.3 ± 1.3^c^	1.6 ± 0.0^a^
ACEPI	0.68 ± 0.04^b^	91.0 ± 0.4^b^	95.0 ± 0.4^b^	94.5 ± 2.1^c^	1.7 ± 0.2^a^

All values are means of triplicates determinations ± standard deviation (SD). Means within the same column with different superscript are significantly different (*p *< .05).

WEPI, water‐extracted pigeon pea protein isolate; MEPI, methanol–extracted pigeon pea protein isolate; ASEPI, ammonium sulfate‐extracted pigeon pea protein isolate; ACEPI, acetone‐extracted pigeon pea isolate; PPF, pigeon pea flour; NB, nitrogen balance; BV, biological value; NPU, net protein utilization; TPD, true protein digestibility; PER, protein efficiency ratio.

## 3 CONCLUSION

The results shows that the techniques used in isolating protein from pigeon pea has improved the quality of the produced protein isolate as a result of drastic reduction and elimination of the inherent antinutrients. The functional properties of the prepared pigeon protein isolate increased significantly when compared with that of flour. Amino acid profiles of pigeon pea isolates are not significantly different from that of pigeon pea flour. The rat assay studies shows that all the protein isolates had good biological value, net protein utilization and true digestibility. Thus, pigeon pea proteins isolated in this study indicates their usefulness as fortificant in a variety of food products to combat protein deficiency in many parts of the world, particularly developing countries.

## 4 CONFLICT OF INTEREST

None declared.
